# Atypical Presentations of Hydroxychloroquine Retinopathy: A Case Series Study

**DOI:** 10.3390/jcm13123411

**Published:** 2024-06-11

**Authors:** Jung Min Lee, Hyeon Yoon Kwon, Seong Joon Ahn

**Affiliations:** Department of Ophthalmology, Hanyang University Hospital, Hanyang University College of Medicine, Seoul 04763, Republic of Korea

**Keywords:** atypical presentations, hydroxychloroquine, retinal toxicity

## Abstract

**Background/Objective:** Hydroxychloroquine retinopathy, traditionally characterized by parafoveal or pericentral outer retinal damage, is explored for atypical presentations in Asian patients. This challenges conventional beliefs regarding onset, retinopathy pattern, and associated visual field defects. **Methods:** Ninety-five patients diagnosed with hydroxychloroquine retinopathy at Hanyang University Hospital underwent screening from January 2010 to December 2023. Swept-source optical coherence tomography (SS-OCT), ultra-widefield fundus autofluorescence (UWF-FAF), and automated visual fields (VF) were employed for detailed structural and functional evaluations. Multifocal electroretinography was performed in selected cases requiring additional objective evidence of retinal toxicity. **Results:** Among 95 patients, 14 (14.7%) exhibited atypical presentations, including very early onset (n = 1), (far) peripheral-dominant damages (n = 4), perivascular involvement (n = 1), bitemporal hemianopsia due to nasal extensive lesions (n = 1), unilateral involvement (n = 2), and asymmetric involvement in retinopathy pattern or severity between the eyes (n = 7). These findings underscore the importance of utilizing expanded imaging techniques, such as ultra-widefield FAF imaging, to identify atypical presentations of retinal involvement. **Conclusions:** Screening physicians should consider these atypical presentations to ensure timely diagnosis and appropriate management in patients undergoing hydroxychloroquine treatment.

## 1. Introduction

Hydroxychloroquine retinopathy, a recognized toxic retinopathy, manifests as paracentral or pericentral outer retinal damage, posing a significant concern for individuals undergoing prolonged hydroxychloroquine treatment [1,2,3,4]. Typically emerging after 5 years of hydroxychloroquine use, this condition is more prevalent in individuals with major risk factors, such as a high daily dose (daily dose/body weight > 5 mg/kg), extended duration of use (beyond 5 years), kidney disease, combined macular disease, and concurrent tamoxifen usage [1]. The American Academy of Ophthalmology (AAO) advocates for regular screening, including optical coherence tomography (OCT), fundus autofluorescence (FAF), automated visual fields (VFs), and/or multifocal electroretinogram (mfERG) after 5 years of hydroxychloroquine use [1,5]. In cases with major risk factors, screening may be initiated earlier to ensure timely detection [1,5].

Typically, hydroxychloroquine retinal toxicity presents as photoreceptor defects in the parafoveal or pericentral areas, with or without accompanying retinal pigment epithelium (RPE) defects on OCT. These alterations correspond to hyper- or hypo-autofluorescence in the parafoveal or pericentral areas on FAF [1,6,7,8,9]. Automated visual fields may reveal patchy scotomas, paracentral ring scotomas, or central islands, contributing to functional evidence of the retinal damage caused by hydroxychloroquine [1,7,9,10]. Furthermore, multifocal electroretinography (mfERG) provides a spatially specific evaluation of retinal function and aids in the comprehensive characterization of hydroxychloroquine-induced retinopathy, which typically shows decreased amplitude in the parafoveal or pericentral regions.

In this case series, we delve into atypical presentations of hydroxychloroquine retinopathy in a cohort of 95 Asian (Korean) patients with hydroxychloroquine retinopathy. Our exploration not only highlights the uniqueness of these cases but also addresses the imperative need to distinguish them from typical presentations of hydroxychloroquine retinopathy. We present a thorough analysis of multimodal imaging, with the overarching goal of contributing to the early detection of atypical presentations of hydroxychloroquine retinopathy, as well as typical manifestations.

## 2. Materials and Methods

### 2.1. Subjects

This study examined 95 patients diagnosed with hydroxychloroquine retinopathy at Hanyang University Hospital between January 2010 and December 2023. Screening for toxicity included OCT, FAF, and VF assessments for all participants, with mfERG utilized in selected cases requiring additional evidence of retinal toxicity. Diagnosis of hydroxychloroquine retinopathy was made based on a combination of abnormalities from at least two of the following tests: OCT, VF, FAF, and mfERG [11,12,13]. The research adhered to the principles outlined in the Declaration of Helsinki and obtained approval from the Institutional Review Board (IRB) at Hanyang University Hospital. Due to the retrospective nature of the study, the IRB waived the requirement for informed consent.

### 2.2. Evaluation

Specifically, to detect structural damage caused by hydroxychloroquine toxicity, we conducted swept-source OCT (SS-OCT) and FAF. SS-OCT, utilizing the DRI-Triton device from Topcon Inc., Tokyo, Japan, involved a 3D macular volume scan covering a 9 × 12-mm^2^ grid centered on the fovea. The scan speed was set at 100,000 A-scans per second, and the axial resolution was 8 µm. Additionally, a 12-mm radial scan was performed, producing 12 radially oriented line scans passing through the fovea, with a scan length of 12 mm, at both baseline and follow-up visits. FAF images were acquired using either an ultra-widefield (UWF) scanning laser ophthalmoscope (Optos 200Tx; Optos PLC, Dunfermline, UK) or a conventional (40°) scanning laser ophthalmoscope (F-10; Nidek, Tokyo, Japan). To assess functional defects, standard automated perimetry, employing the 30–2, 10–2, or both strategies, was conducted using a Humphrey Field Analyzer II or III (Carl Zeiss Meditec, Dublin, CA, USA). Additionally, mfERG was performed in selected cases requiring additional objective evidence of retinal toxicity based on the 61-hexagon stimulus pattern of the VERIS Clinic system (Electro-Diagnostic Imaging, Inc., Redwood, CA, USA) according to the guidelines of the International Society for Clinical Electrophysiology of Vision (ISCEV).

Based on the primary site of retinal damage, eyes affected by hydroxychloroquine retinopathy were categorized into distinct groups: parafoveal (involving photoreceptor/RPE disruption within 2–8° of the fovea), pericentral (exhibiting outer retinal damage beyond 8° from the fovea), or mixed (displaying similar or extensive involvement in both areas) [1]. The severity of retinopathy in these eyes was classified as early (localized hyper-autofluorescence on FAF and/or localized photoreceptor defects without RPE involvement on OCT), moderate (involving photoreceptor damage [hyper-autofluorescence] with a partial [>180°] or complete ring on FAF), or severe (manifesting as combined RPE damage represented by hypo-autofluorescence on FAF) [1,14]. The determination of severity was primarily based on FAF, with the addition of OCT in cases where FAF abnormalities were inconclusive or unidentified.

### 2.3. Definitions and Analysis

**Figure 1:**
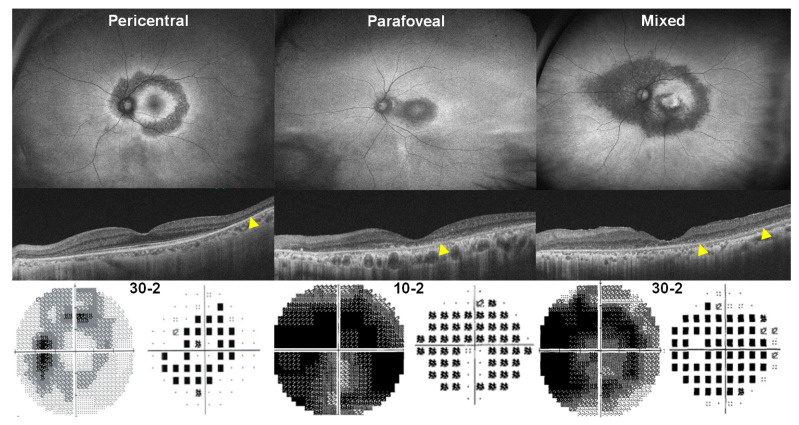
Illustration of typical cases demonstrating pericentral, parafoveal, and mixed patterns of hydroxychloroquine retinopathy in 63-, 69-, and 65-year-old Korean women who have been taking hydroxychloroquine for 7, 24, and 20 years, respectively. **Top**: fundus autofluorescence images; **middle**: optical coherence tomography (OCT) scans; **bottom**: grayscale (**left**) and pattern deviation (**right**) plots of Humphrey visual fields. Yellow arrowheads indicate outer retinal defects on OCT in the areas of damage (parafoveal, pericentral, or mixed).

The structural and functional findings, along with clinical characteristics, in typical cases are summarized in Table 1. Typical cases, as defined in the literature, exhibit parafoveal or pericentral (or both) photoreceptor defects with or without accompanying retinal pigment epithelium (RPE) defects on OCT in both eyes [1]. These manifestations typically occur after 5 years of hydroxychloroquine use. Additionally, these cases exhibited parafoveal- or pericentral-dominant hyper- or hypo-autofluorescence on FAF, along with corresponding visual field defects, as illustrated in Figure 1. The patterns (parafoveal or pericentral) or severities (early, moderate, or severe) of retinal damage are typically symmetric in both eyes.

In instances where patients exhibited atypical presentations of hydroxychloroquine retinopathy involving significant variations in disease onset and structural/functional abnormalities, findings from multimodal imaging and functional modalities, including OCT, FAF, VF, and mfERG, were thoroughly evaluated and documented. A comprehensive comparison of each atypical case with typical presentations of hydroxychloroquine retinopathy was tabulated.

A descriptive statistical analysis was performed for the demographic data, details of hydroxychloroquine use, and clinical characteristics of retinopathy. The prevalence of each atypical presentation was also investigated. Statistical analyses were performed using SPSS software (version 27.0; IBM Corp.: Armonk, NY, USA).

## 3. Results

Among the 95 patients diagnosed with hydroxychloroquine retinopathy, atypical presentations were observed in 14 (14.7%) cases. Of these, seven notable cases were selected for detailed descriptions and presentations. Table 2 provides a summary of these presentations, including instances of very early onset of the disease (Case 1), (far) peripheral-dominant retinal damages (Cases 2 and 3), perivascular involvement (Case 4), bitemporal hemianopsia due to nasal half-ring lesion (Case 5), asymmetric involvement in terms of pattern (Case 6) and severity (Case 7).

### 3.1. Early Presentation/Onset (within 1 Year)

**Figure 2:**
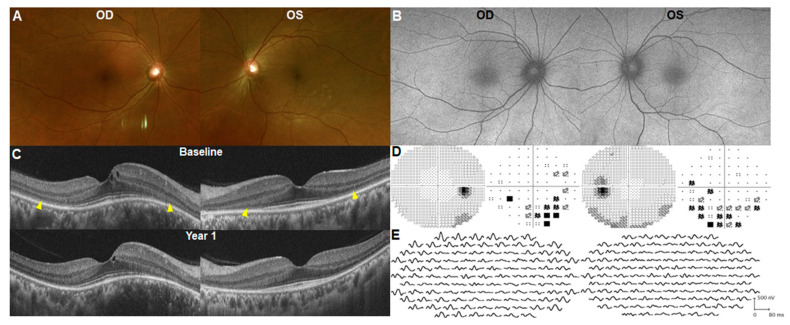
Fundus photograph (**A**), fundus autofluorescence (**B**), optical coherence tomography (OCT; (**C**)), Humphrey 30-2 visual field (HVF) results (**D**), and multifocal electroretinogram (ERG; (**E**)) of Case 1 with 6-month-onset hydroxychloroquine retinopathy. Although the fundus photograph and autofluorescence images show normal findings, OCT and HVF show characteristic paracentral photoreceptor loss ((**C**), indicated by arrowheads) and scotoma (**D**) in both eyes. Multifocal ERG also shows a decrease in amplitude in the paracentral areas (**E**).

A 44-year-old Korean woman, weighing 56 kg, underwent baseline HCQ retinopathy screening. She had received 300 mg HCQ daily for 6 months for rheumatoid arthritis (RA) and reported progressive loss of peripheral visual field and light flashes for 1 month. She had no other medical conditions except for rheumatic disease, and her family history did not indicate retinal diseases. The initial systemic workup by the rheumatologist was unremarkable. Her BCVA was 20/20 in both eyes and slit-lamp examination revealed no abnormalities. Dilated fundus examination and FAF (Figure 2A,B) showed a normal retina. However, OCT revealed photoreceptor loss in the paracentral areas of both eyes (Figure 2C). The 30-2 strategy of automated perimetry identified paracentral scotomas in both eyes (Figure 2D). Multifocal ERG demonstrated a generalized depression of amplitude and delayed latency in the paracentral area (Figure 2E). Discontinuation of the drug did not cause further progression of symptoms or damage to the photoreceptor over 1 year (Figure 2C, bottom). The early onset of disease within 1 year of HCQ use was noted in only one (1.1%) out of 95 patients.

### 3.2. Nasal Peripheral Involvement

**Figure 3:**
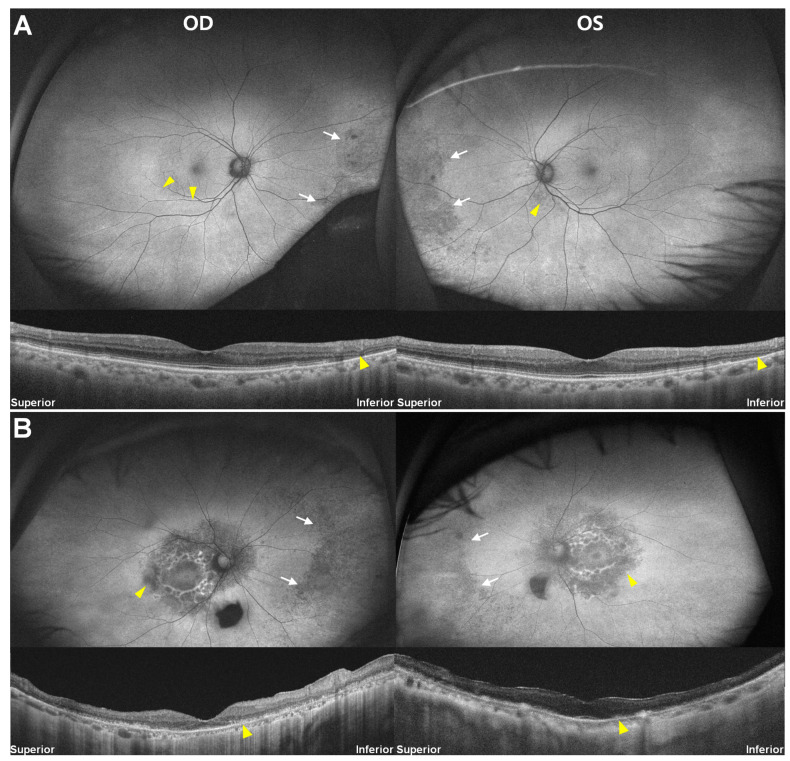
Fundus autofluorescence (**top**) and optical coherence tomography images (**bottom**) of Cases 2 (**A**) and 3 (**B**). Yellow arrowheads indicate characteristic (parafoveal or pericentral) damage, whereas white arrows indicate nasal (far) peripheral hypo-autofluorescence, an atypical presentation of hydroxychloroquine retinopathy.

A 72-year-old woman undergoing hydroxychloroquine treatment with a daily dose of 200 mg for 25 years for RA (Case 2) underwent hydroxychloroquine retinopathy screening. UWF-FAF (Figure 3A) revealed subtle hyper-autofluorescence around the right eye’s inferior vascular arcade, equivocal peripapillary hypo-autofluorescence in the left (arrowheads), and bilateral circumferential hypo-autofluorescence in the nasal periphery (arrows). However, OCT confirmed characteristic photoreceptor loss in the inferior pericentral areas (arrowheads). Figure 2B depicted more severe and extensive damage than Figure 3A, but with a similar distribution of far peripheral and posterior pole lesions, in another 75-year-old woman with hydroxychloroquine retinopathy, taking a daily dose of 200 mg for 20 years (Case 3). UWF-FAF (B) showed hypo-autofluorescence over the nasal far periphery and posterior pole (arrowheads) bilaterally, corresponding to photoreceptor and retinal pigment epithelium (RPE) defects on OCT (arrows). Similar to the case in Figure 3A, both eyes exhibited a relatively intact retina with normal autofluorescence between the far peripheral and posterior pole lesions. Peripheral dominance was noted in four of 95 (4.2%) patients in our cohort of hydroxychloroquine retinopathy.

### 3.3. Perivascular Involvement

**Figure 4:**
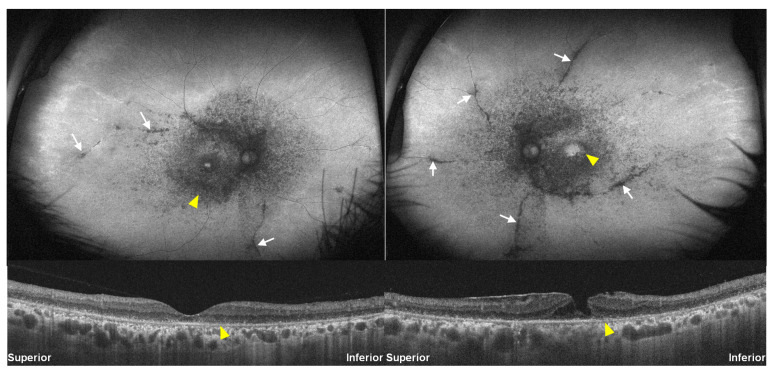
Fundus autofluorescence (**top**) and optical coherence tomography images (**bottom**) of Case 4 with perivascular involvement, which is more prominent in the left eye. Yellow arrowheads indicate characteristic (parafoveal or pericentral) damage, whereas white arrows indicate perivascular hypo-autofluorescence.

A 60-year-old woman on hydroxychloroquine treatment, with a daily dose of 300 mg for systemic lupus erythematosus for 20 years (Case 4; Figure 4), exhibited perivascular hypo-autofluorescence (white arrows) in the peripheral retina, along with characteristic parafoveal and pericentral hypo-autofluorescence on FAF and outer retinal defects on OCT (arrowheads). This case highlighted atypical hydroxychloroquine retinopathy affecting perivascular areas in the peripheral retina. The unusual presentation of perivascular involvement was noted in only one patient (1.1%).

### 3.4. Bitemporal Hemianopsia Due to Nasal Extensive Lesion

**Figure 5:**
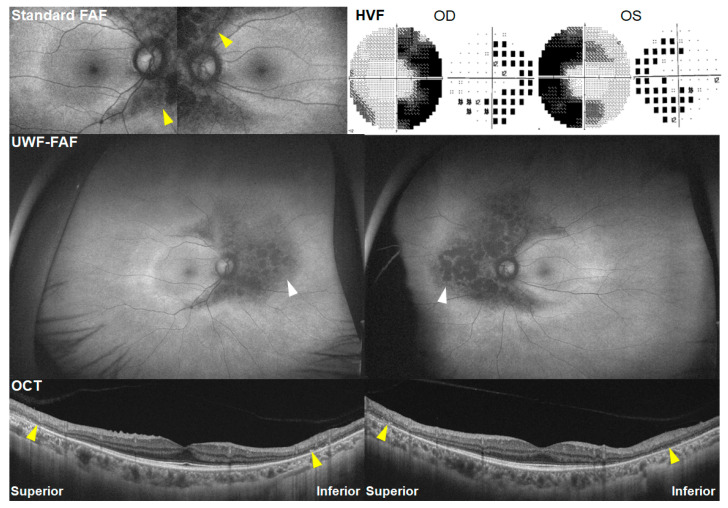
Conventional 40° (**top left**) and ultra-widefield fundus autofluorescence (UWF-FAF), Humphrey 30-2 visual field test (HVF; **top right**), and optical coherence tomography images (**bottom**) of Case 5 with pericentral retinopathy (yellow arrowheads) showing bitemporal hemianopsia sparing the central field on HVF and nasal peripheral extension (white arrowheads) on UWF-FAF.

The 54-year-old female in Figure 5, who had been using HCQ for almost 6 years for Sjögren’s syndrome, underwent standard 40° FAF (Figure 5, top left) and Humphrey visual field tests (Figure 5, top right). These tests revealed peripapillary and pericentral hypo-autofluorescence (yellow arrowheads) and bitemporal hemianopsia sparing the central field on grayscale (left) and pattern deviation (right) plots of Humphrey 30-2 tests in both eyes, respectively. Brain imaging was performed to identify potential neurological causes of bitemporal hemianopsia, which yielded no abnormal findings. However, UWF-FAF imaging indicated nasal peripheral hypo-autofluorescence of a half-ring shape in both eyes, corresponding well with temporal hemianopsia in both eyes. Although bitemporal hemianopsia was noted in only one (1.1%) patient, a similar lesion, pericentral and nasally extensive, was observed in six of 95 patients (6.3%).

### 3.5. Unilateral Involvement

**Figure 6:**
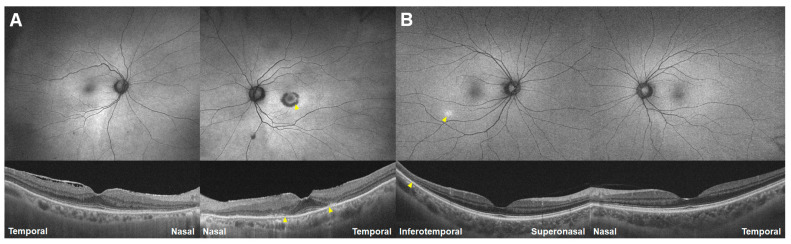
Fundus autofluorescence (**top**) and optical coherence tomography images (**bottom**) in two patients (**A** [Case 6] and **B**) with unilateral involvement of retinopathy. Yellow arrowheads indicate characteristic (parafoveal or pericentral) damage.

A 75-year-old female patient (Case 6), with a history of RA spanning 23 years and ongoing HCQ use, is depicted in Figure 6A. Utilizing FAF and OCT, the left eye exhibited parafoveal ring-shaped hypo-autofluorescence on FAF (Figure 6A, right panel) and a parafoveal outer retinal defect on OCT, graded as severe HCQ retinopathy, while the right eye displayed only photoreceptor attenuation with no definite defect or loss in the photoreceptor layers on OCT and no definite abnormality on FAF (Figure 6A, left). Another case in Figure 6B showed an asymmetric pattern of retinopathy as pericentral photoreceptor loss on the inferotemporal area, corresponding to focal hyper-autofluorescence (arrowheads), in the right eye and no identifiable abnormality in the left. This atypical presentation, unilateral hydroxychloroquine retinopathy, was identified in two of 95 patients (2.1%).

### 3.6. Asymmetric Involvement in Retinopathy Severity/Pattern

**Figure 7:**
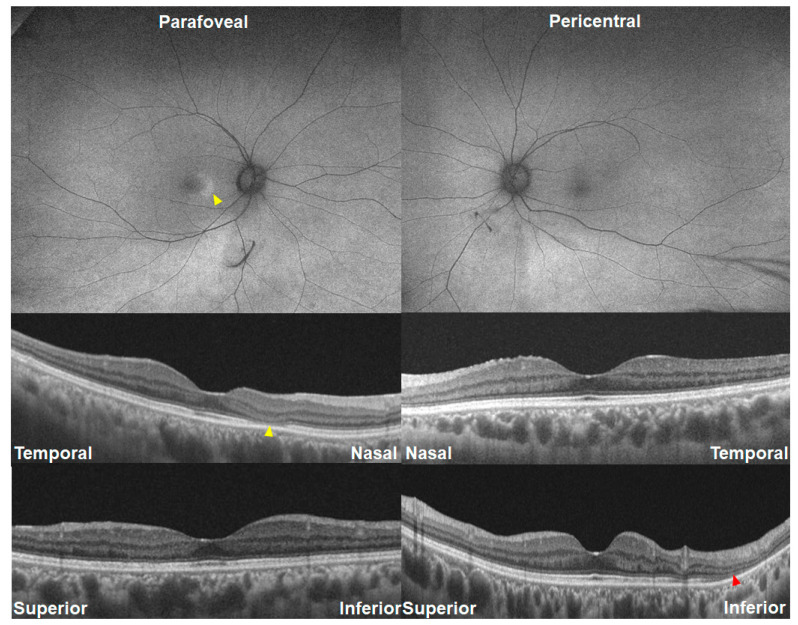
Fundus autofluorescence (FAF; **top**) and optical coherence tomography images (OCT; **bottom**) of Case 7 with asymmetric involvement of retinopathy pattern. The right eye shows parafoveal hyper-autofluorescence on FAF and loss of the parafoveal ellipsoid zone line on OCT (parafoveal retinopathy as indicated by yellow arrowheads), whereas the left demonstrates inferior pericentral photoreceptor loss (pericentral retinopathy as indicated by red arrowhead).

**Figure 8:**
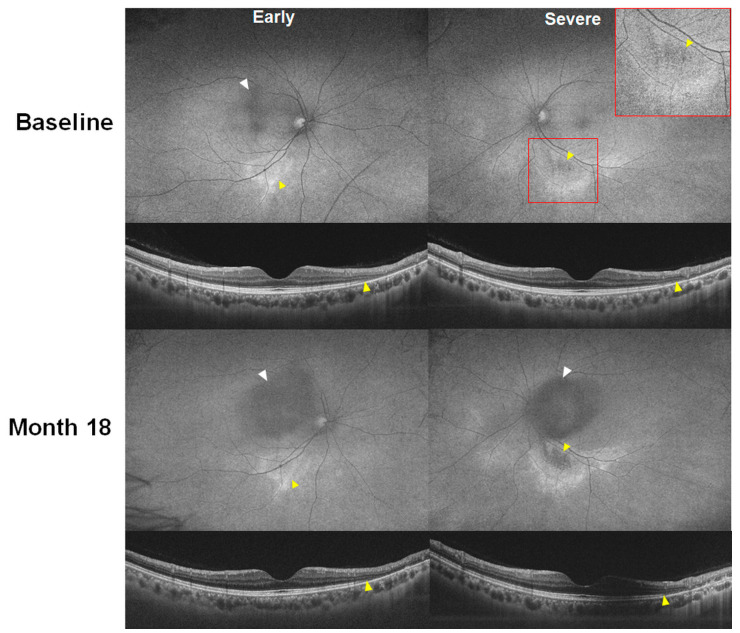
Fundus autofluorescence (FAF; **top**) and optical coherence tomography (OCT; **bottom**) images of Case 7 with asymmetric involvement of retinopathy severity at baseline and 18-month follow-up visits. The right eye shows localized hyper-autofluorescence on FAF and localized photoreceptor loss on OCT (both indicated by yellow arrowheads), indicating early retinopathy. The left eye demonstrates mild hypo-autofluorescence, more distinguishable in the magnified image (inset in the upper right corner), and localized outer retinal defects on OCT (yellow arrowheads). At month 18, the right eye shows no progression, whereas the left eye reveals retinopathy progression with more extensive photoreceptor loss on OCT and definite hypo-autofluorescence on FAF (both indicated by yellow arrowheads). White arrowheads indicate artifacts due to cataract progression.

Figure 7 and Figure 8 demonstrate photographic examples showing asymmetric involvement in the severity or pattern of hydroxychloroquine retinopathy. Figure 7 shows a case of parafoveal retinopathy in the right eye and pericentral retinopathy in the left eye in a 42-year-old female (Case 7) taking 200 mg of hydroxychloroquine for 12 years. Figure 8 shows baseline and 18-month follow-up images of fundus autofluorescence and OCT, showing the early stage in the right and the severe stage in the left. The left eye shows hypo-autofluorescence (arrowhead) around the inferior vascular arcade at baseline, leading to more extensive and definite hypo-autofluorescence 18 months later. In contrast, the right eye with early retinopathy showed no progression of the photoreceptor defect or hyper-autofluorescence. The difference in retinopathy progression between the two eyes seems to have originated from the difference in retinopathy severity.

Among the 95 patients with hydroxychloroquine retinopathy, asymmetric involvement either in retinopathy pattern (parafoveal or pericentral) or severity (early, moderate, or severe) was noted in seven (7.4%) patients. Specifically, asymmetry in retinopathy pattern was noted in six (6.3%) patients whereas that in retinopathy severity was observed in two (2.1%).

Table 3 summarizes the atypical presentations of the aforementioned cases and their relative prevalences, along with appropriate screening methods for detection.

## 4. Discussion

Hydroxychloroquine retinopathy is a well-established concern, with its typical manifestations widely recognized. Previous findings emphasize the importance of regular ophthalmological monitoring, particularly in patients undergoing long-term hydroxychloroquine therapy, to detect any potential retinal changes at early stages and mitigate the risk of irreversible damage [15,16]. However, our case series reports atypical presentations of hydroxychloroquine retinopathy in Asian (all Korean) patients. The outlined atypical presentations in this study include very early onset of disease, (far) peripheral-dominant retinal damage, perivascular involvement, bitemporal hemianopsia due to nasal extensive lesions, unilateral involvement, and asymmetricity between the eyes in retinopathy severity and pattern. These presentations deviate from the disease onset and conventional areas seen in typical cases of hydroxychloroquine retinopathy.

In our case series, a strikingly early onset of hydroxychloroquine retinopathy was observed in a 44-year-old patient within just 6 months of drug use. This early-onset toxicity, which has been recently reported [17,18], challenges the conventional belief that toxic retinal effects typically manifest after at least 5 years of hydroxychloroquine use [1]. The rapid onset raises concerns about the current screening recommendations, which suggest performing baseline screening within 1 year using fundus examination for pre-existing macular conditions and beginning annual screening from 5 years of use [1,16], because following this recommendation might have led to a very advanced stage at diagnosis in Case 1.

Another atypical presentation is the dominance of retinal damage in the (far) peripheral regions rather than the parafoveal or pericentral areas. This peripheral dominance challenges the established understanding of hydroxychloroquine retinopathy as a predominantly central pathology [6,19]. The observed peripheral involvement underscores the importance of comprehensive retinal imaging over the whole retina, such as ultra-widefield imaging, to capture these unusual manifestations [14]. Our series also includes a case demonstrating perivascular involvement in the peripheral retina, a presentation not previously reported for hydroxychloroquine retinopathy. This finding suggests that the toxic effects of hydroxychloroquine might extend beyond the photoreceptor and retinal pigment epithelium, affecting the peripheral retina adjacent to vascular structures, and highlights the vulnerability of perivascular areas to hydroxychloroquine-induced retinal toxicity. Recognition of such atypical presentations is crucial for a thorough understanding of the disease spectrum and for close monitoring and identification of retinal damage.

Bitemporal hemianopsia due to nasal extensive lesions, as illustrated in one of our cases, emphasizes the potential impact of hydroxychloroquine retinopathy on visual fields and necessitates a careful differential diagnosis with neurological visual field defects. This manifestation might lead to misdiagnosis, as the symptoms could be erroneously attributed to neurological causes [20]. Awareness of this atypical presentation is essential for clinicians, highlighting the need for a comprehensive retinal evaluation, including the nasal periphery, when HCQ users present with visual field defects.

Another noteworthy, atypical presentation observed was unilateral involvement, as illustrated by Case 6. Despite both eyes being exposed to hydroxychloroquine, significant retinal changes were only observed in the left eye. The underlying condition predisposing the left eye to retinopathy remains unclear. It is possible that the other eye may have subclinical involvement, necessitating long-term follow-up to identify characteristic changes. Such unilateral presentations present diagnostic challenges, as outer retinal changes are typically associated with unilateral conditions such as central serous chorioretinopathy and other macular degenerative diseases.

Furthermore, our report identified cases with asymmetric involvement in retinopathy severity or pattern, exemplified in Figure 7 and Figure 8. Notably, these patients displayed parafoveal retinopathy in the right eye and pericentral retinopathy in the left eye, demonstrating a discrepancy in the distribution or different progression of retinal changes between the two eyes. These findings underscore the heterogeneity of HCQ retinopathy and suggest potential variations in disease mechanisms or susceptibility between the eyes. The observation of asymmetry in retinopathy severity and pattern highlights the importance of individualized assessment and monitoring strategies for each eye (e.g., 10-2 for the eye with parafoveal retinopathy and wider tests for the other eye with pericentral retinopathy) in patients receiving HCQ therapy, considering the possibility of differential involvement and progression between the eyes.

The identification of these atypical presentations raises important considerations for screening and diagnosis. The current guidelines, which primarily emphasize the detection of parafoveal or pericentral pathology that typically occurs after 5 years of use, may not be sufficient to detect these atypical presentations of hydroxychloroquine retinopathy [1]. Our findings underscore the need for a more comprehensive approach to whole retinal screening, incorporating ultra-widefield FAF imaging, given its ability to capture peripheral changes, suggesting its usefulness in identifying uncommon presentations in hydroxychloroquine retinopathy [14].

This study has certain limitations, including its retrospective nature and the small number of atypical cases identified in our evaluation of atypical presentations from a relatively large cohort of patients with hydroxychloroquine retinopathy (n = 95). Further research with a larger sample size is warranted to validate our findings and explore additional atypical presentations. Additionally, the ethnic background (Asians) contributing to the variations in presentation patterns of hydroxychloroquine retinopathy is crucial for understanding our cases, as pericentral cases are more prevalent in Asian patients than in other ethnic groups [1]. Furthermore, Case 1 may have a pre-existing inherited retinal disease, such as retinitis pigmentosa. However, we performed an NGS panel covering approximately 300 genes associated with inherited retinal diseases for the patient, as we suspected this possibility, although she denied any family history of retinal diseases. The testing revealed no pathogenic or likely pathogenic variants. Furthermore, she had slowly progressive visual decline with field constriction while on the drug, whereas she experienced no further progression after drug cessation for one year. Nevertheless, we cannot completely exclude the possibility of such retinal dystrophy, which should be carefully assessed and distinguished from hydroxychloroquine retinopathy, particularly in cases with short-term use.

In conclusion, our case series reports the atypical presentations of hydroxychloroquine retinopathy in Asian patients, suggesting variable presentations of hydroxychloroquine retinopathy that clinicians should be aware of. Furthermore, screening physicians need to be vigilant in retinopathy screening for the possibility of early-onset disease, peripheral dominance, perivascular involvement, and unusual visual field defects.

## Figures and Tables

**Table 1:** Presentations of typical cases of hydroxychloroquine retinopathy in the literature.

Characteristics	Typical Case
Sex	Typically women
Duration of hydroxychloroquine use	Typically occur after 5 year’s use
Structural findings	Photoreceptor defects and/or retinal pigment epithelium defects on OCT or hyper- or hypoautofluorescence on FAF in the parafoveal or pericentral areas
Functional (perimetric) findings	Paracentral patchy/ring scotoma, central island
Eye involvement	Bilateral, symmetric

FAF = fundus autofluorescence; OCT = optical coherence tomography.

**Table 2:** Presentations of typical cases with hydroxychloroquine (HCQ) retinopathy in the literature and summary of seven cases with atypical presentations.

Characteristics	Case 1	Case 2	Case 3	Case 4	Case 5	Case 6	Case 7
Age/sex	44/F	72/F	75/F	60/F	54/F	75/F	39/F
Medical diagnosis for HCQ use	RA	RA	SLE	SLE	Sjogren’s syndrome	SLE	SLE
Daily dose of hydroxychloroquine/body weight ratio	5.4 mg/kg	3.3 mg/kg	3.0 mg/kg	5.6 mg/kg	4.2 mg/kg	8.0 mg/kg	4.0 mg/kg
Duration of hydroxychloroquine use	**6 months**	25 years	20 years	20 years	6 years	23 years	8 years
Structural findings	Photoreceptor defects on pericentral areas	Hypo-AF on **nasal far periphery** and inf. pericentral photoreceptor defect	Hypo-AF on **nasal far periphery** and posterior pole	**Perivascular** hypo-AF	Half-ring-shaped nasal peripheral hypo-AF	Ring-shaped parafoveal **hypo-AF only in the left eye**	Localized pericentral hyper-AF (early stage) in the right and hypo-AF in the left (severe stage)
Functional (perimetric) findings	Paracentral scotoma	Patchy scotoma	Central island	Ring scotoma	**Bitemporal hemianopsia** with central sparing	Ring **scotoma only in the left eye**	Sup. patchy scotoma
Symmetricity between the two eyes	Symmetric in both pattern and severity	Symmetric	Symmetric	Symmetric	Symmetric	**Asymmetric (unilateral involvement)**	**Asymmetric in severity**

Boldface indicates atypical presentations. RA = rheumatoid arthritis; SLE = systemic lupus erythematosus; AF = autofluorescence.

**Table 3:** Atypical presentations of hydroxychloroquine retinopathy and appropriate methods for detection.

Atypical Presentations	Figure No.	Prevalence, n (%)	Methods for Detection
Early disease onset	Figure 2	1 (1.1%)	Baseline screening performed using OCT within 1 year of hydroxychloroquine use
Far peripheral involvement (dominance)	Figure 3	4 (4.3%)	Widefield FAF
Perivascular involvement	Figure 4	1 (1.1%)	Widefield FAF
Bitemporal hemianopsia due to nasal extensive lesion	Figure 5	1 (1.1%)	Automated visual field, widefield FAF
Unilateral involvement	Figure 6	2 (2.1%)	Standard screening modalities *
Asymmetric involvement	Figure 7 and Figure 8	7 (7.4%)	
in pattern of retinopathy	Figure 7	6 (6.3%)	Widefield OCT or FAF (for detection of pericentral pattern in one eye)
in severity of retinopathy	Figure 8	2 (2.1%)	Standard screening modalities *

* OCT, FAF, visual fields, mfERG.

## Data Availability

Data and materials can be requested by e-mail and will be provided after consultation with the IRB.
